# 3D Micro/Nanopatterning of a Vinylferrocene Copolymer

**DOI:** 10.3390/molecules25102438

**Published:** 2020-05-23

**Authors:** Dennis Löber, Subhayan Dey, Burhan Kaban, Fabian Roesler, Martin Maurer, Hartmut Hillmer, Rudolf Pietschnig

**Affiliations:** 1 Institute of Nanostructure Technologies and Analytics (INA) and Center for Interdisciplinary Nanostructure Science and Technology (CINSaT), University of Kassel, Heinrich-Plett-Straße 40, 34132 Kassel, Germany; 19.denn.92@googlemail.com (D.L.); kaban@ina.uni-kassel.de (B.K.); 2 Institute of Chemistry and Center for Interdisciplinary Nanostructure Science and Technology (CINSaT), University of Kassel, Heinrich-Plett-Straße 40, 34132 Kassel, Germany; subhayan_dey@uni-kassel.de (S.D.); fabian.roesler@uni-kassel.de (F.R.); maurer@uni-kassel.de (M.M.)

**Keywords:** vinylferrocene, copolymer, methyl methacrylate, reverse nanoimprint lithography

## Abstract

In nanoimprint lithography (NIL), a pattern is created by mechanical deformation of an imprint resist via embossing with a stamp, where the adhesion behavior during the filling of the imprint stamp and its subsequent detachment may impose some practical challenges. Here we explored thermal and reverse NIL patterning of polyvinylferrocene and vinylferrocene-methyl methacrylate copolymers to prepare complex non-spherical objects and patterns. While neat polyvinylferrocene was found to be unsuitable for NIL, freshly-prepared vinylferrocene-methyl methacrylate copolymers, for which identity and purity were established, have been structured into 3D-micro/nano-patterns using NIL. The cross-, square-, and circle-shaped columnar structures form a 3 × 3 mm arrangement with periodicity of 3 µm, 1 µm, 542 nm, and 506 nm. According to our findings, vinylferrocene-methyl methacrylate copolymers can be imprinted without further additives in NIL processes, which opens the way for redox-responsive 3D-nano/micro-objects and patterns via NIL to be explored in the future.

## 1. Introduction

Ferrocene-containing polymers are among the most prominent examples of metallopolymers and have been reviewed under various studies [1,2,3,4,5,6,7,8]. Many of the numerous applications emerge from the redox-activity of the ferrocene unit, ranging from switchable surface polarity [9] or catalytic activity [10] to tunable photonic [11] or contractible [12] materials to name just a few. However, diamagnetic non-oxidized ferrocene-based polymers and block-co-polymers show equally attractive features, with particular achievements in supramolecular assembly and information encoding [13].

Nanoimprint lithography (NIL) is a powerful time and cost-efficient method for the preparation of reproducible and uniform patterns and structures. NIL processes employing polymeric materials have been well established in recent years since seminal studies in 1996 [14,15,16,17,18,19,20]. Nevertheless, many imprint procedures rely on the use of standardized polymer mixtures requiring additives to ensure proper structure replication. To overcome such limitations, the NIL procedure was further improved, which recently allowed for the incorporation of nanoparticles and luminescent molecules into imprinted structures [17,21,22]. Ferrocene-based polymers have rarely been used as imprint resists, but a few seminal reports describe the use of polymers with ferrocene in the main chain or as a pendant unit [23,24,25,26,27]. Here, we present our results of structuring previously described vinylferrocene-methyl methacrylate copolymers into three-dimensional micro/nano-patterns using the NIL procedure [28]. The well-defined micro/nanostructuring of this material would be an important step for the equipment of complex non-spherical objects with potential redox responsivity.

## 2. Results and Discussion

The primary challenge for this work remained in the proper choice of optimum ferrocene-containing air-stable polymers which could be dissolved in a reasonably high-boiling solvent (like toluene), crucial for the subsequent lithographic process. Due to their limited solubility in common organic solvents, except tetrahydrofuran, neat polyferrocenylsilanes, polyvinylferrocene, and stoichiometric co-polymers of vinylferrocene and methyl methacrylate (MMA) (vinylferrocene: MMA, 1:1), despite being air and moisture stable, were found unsuitable for our purposes. However, co-polymers of vinylferrocenes and stoichiometric excess MMA (vinylferrocene: MMA, 1:2) were found soluble in common organic solvents, and therefore, 10% solutions of these polymers in toluene have been used for the further lithographic work. Here we focus our report on the co-polymer of vinylferrocene (VFc) and stoichiometric excess methyl methacrylate (MMA) prepared from a (VFc: MMA) ratio of 1:2, as this showed the best imprint results in our study.

Vinylferrocene copolymers have been prepared by radical polymerization technique using a catalytic amount of azobisisobutyronitrile (AIBN) from VFc, co-polymerized with MMA, following an established optimized procedure [28,29]. Monovinylferrocene had been synthesized from ferrocenecarboxaldehyde starting from ferrocene [30,31]. The identity and purity of the resulting poly[(methyl methacrylate)-co-(vinyl ferrocene)] (MVF) were established using ^1^H- and ^13^C-NMR and elemental analysis. Unanimously, the analytical results indicate a (VFc: MMA) ratio of approximately 1:3 in the co-polymer despite a 1:2 ratio of the reactants during polymer synthesis. The average out of four runs shows a low standard deviation of 1: 2.8(1) based on ^13^C-NMR using inverse gated decoupling for measurements without NOE.

### 2.1. Fabrication of Master Template

**Figure 1:**
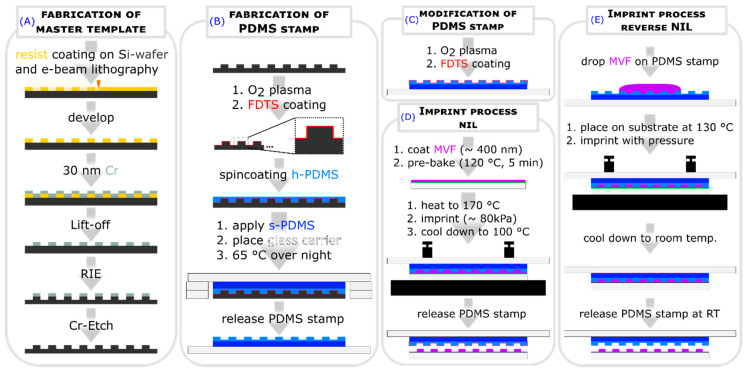
Schematic representation of the fabrication of the master template (**A**), casting process of our hybrid PDMS stamp (**B**), modification of the hybrid PDMS stamp (**C**) for the thermal NIL imprint (**D**) and the reverse NIL process (**E**), which was used to fabricate MVF-imprinted structures. Perfluorodecyltrichlorosilane (FDTS) serves as an anti-adhesive layer (cf. Section 3.3).

In order to use NIL for imprinting structures with our co-polymer, a master template was fabricated with the periodic cross, square, and circular structures with diameters and side lengths of 3 µm, 1 µm, 545 nm, 506 nm and a height of 525 nm using e-beam lithography. Casting the master template with h- and s- PDMS resulted in a hybrid PDMS stamp (see Figure 1A,B).For the fabrication of the structures, we used two NIL methods, the thermal NIL with a modified PDMS stamp (see Figure 1C,D) and the reverse NIL (see Figure 1E).

### 2.2. Surface Structuring by Thermal NIL

**Figure 2:**
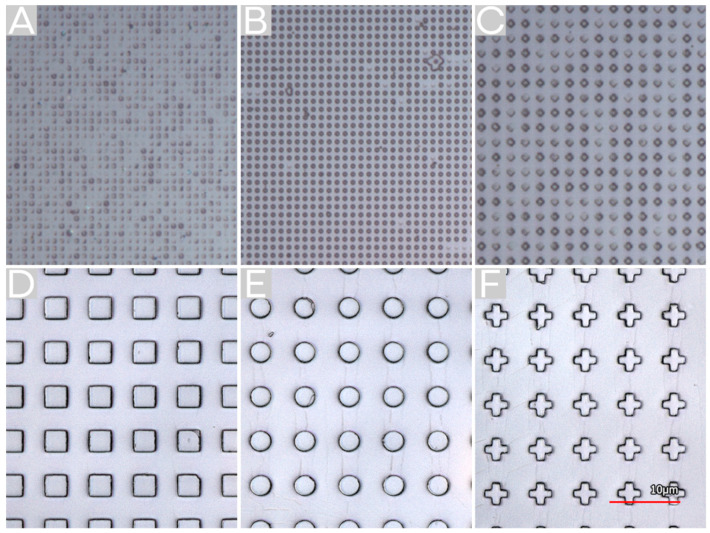
Imprinted patterns fabricated by thermal NIL. Squares with a 506 nm side length (**A**), circles with a diameter of 542 nm (**B**), crosses with a side length of 1 µm (**C**), and squares (**D**), circles (**E**), and crosses (**F**) with side length diameters of 3 µm, respectively.

A solution of poly [(methyl methacrylate)-co-(vinyl ferrocene)] (MVF) in toluene was used to coat an approximately 400 nm thick layer of MVF on a glass substrate by spin coating (Figure 1D). Then, the coated glass carrier was pre-baked to evaporate the remaining toluene to avoid swelling of the used hybrid PDMS stamp. Afterward, this layer was structured by thermal NIL. The fabricated patterned surfaces show a good structure transfer for the 3 µm structures (Figure 2D–F). For structures with smaller feature sizes, the pattern transfer was incomplete (Figure 2A,C). The quality of the imprinted structures decreases with decreasing feature size. Additionally, the quality decreases from circles, which shows a good pattern transfer, also for a feature size of 546 nm (Figure 2B), over squares to crosses, with a poor pattern quality for structures with a feature size of 1 µm. For the structures of 3 µm feature size, a pattern height of approximately 500 nm was determined. Since the residual layer is negligible for our investigations, it was not considered. The imprinted structures exhibit side lengths/diameters of 3.3 µm, 3.0 µm, and 3.3 µm for squares, circles, and crosses, respectively. This discrepancy of the pattern size in comparison with the master of approximately 10% could be a result of flowing of the co-polymer after demolding. By contrast, the separation of the mold and polymer layer at lower temperatures resulted in more damaged patterns, and therefore, a higher demolding temperature was preferred.

### 2.3. Surface Structuring by Reverse NIL

**Figure 3:**
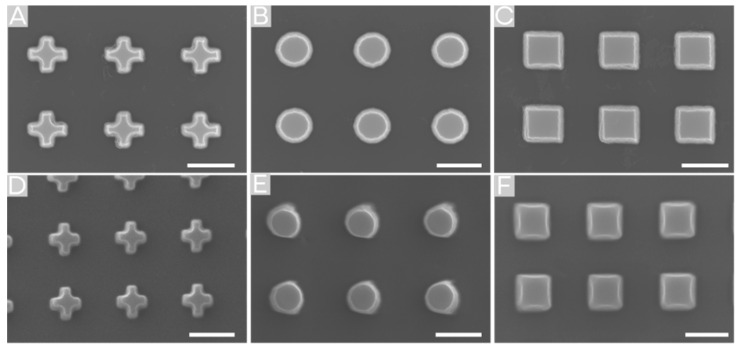
Comparison of the master template (**A**–**C**) with the imprint results of 506 nm feature size structures imprinted via reverse NIL (**D**–**F**). A slight change in structure size is visible. The scale bar indicates 500 nm.

In contrast to thermal NIL, imprinting the structures via reverse NIL resulted in higher shape stability and better transfer quality, whereby the size has a shrinkage of nearly 10% for structures of small feature size (542 nm and 506 nm), but not for 3 µm and 1 µm. Additionally, there are no differences between the different shapes. In Figure 3D–F, imprinted structures (506 nm) via reverse NIL are shown in comparison to the silicon master template (Figure 3A–C).

### 2.4. Comparison of Fabricating MVF Structured Surfaces via Thermal NIL vs. Reverse NIL

**Figure 4:**
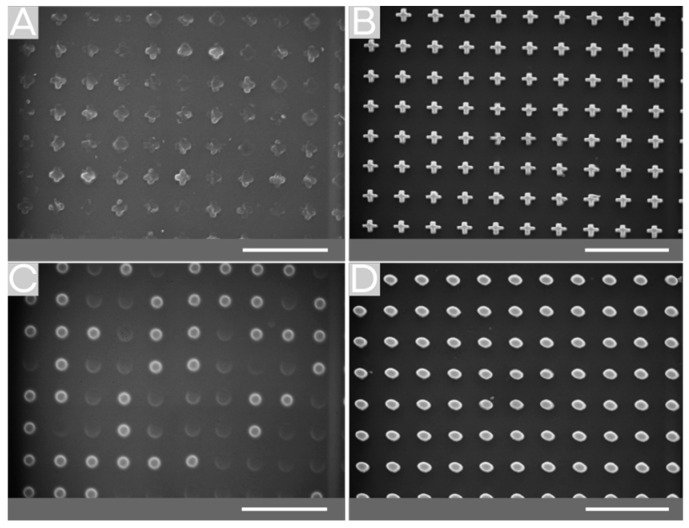
Comparison of thermal and reverse NIL imprinted structures. The improperly-imprinted area observed for thermal NIL (**A**,**C**), whereby the imprint via reverse NIL structures in sub-micron dimensions can be imprinted properly (**B**,**D**). The scale bar indicates 3 µm.

The evaluation of the imprint methods regarding the structuring of MVF shows that the reverse NIL is more suitable for the fabrication of smaller structures than the thermal NIL (see Figure 4B,D). Another advantage of this method is its higher speed and lower temperature. Depending on the requirements, the minor feature of this method, which is that the residual layer forms only at the point where the drop is located, can be an advantage. The major advantage of thermal NIL is that the substrate surface is coated homogeneously over the whole surface. These coatings are better suited for further investigation of electrochemical oxidation.

### 2.5. Redox Behavior of MVF

**Figure 5:**
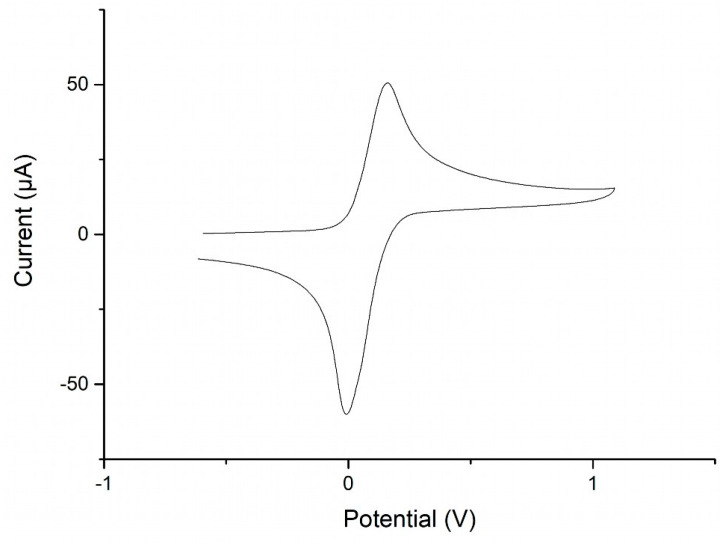
Cyclic voltammogram of MVF in dichloromethane (referenced vs. FcMe_10_/FcMe_10_^+^), voltage sweep 250 mV/s.

The redox properties of MVF have been explored via cyclic voltammetry (CV) in dichloromethane solution. The CV curves show oxidation of the ferrocenyl units at +159 mV and reduction at −10 mV (both vs. FcMe_10_/FcMe_10_^+^) and the well-structured waves show no indication for electronic coupling or interaction between the iron centers (Figure 5). This behavior is along the expected lines for polymers with spatially-separated pendant ferrocene units [6,32]. The separation of the peak potentials indicates quasi-reversible characteristics of the MVF polymer, and the oxidation peak potential at 159 mV implies that under ambient conditions oxidation by air is not to be expected. In line with this, handling and manipulation of solutions and films of MVF show no visible changes in air. Oxidation of films using iodine solution as an oxidant, having a more anodic oxidation potential, results in some color changes for patchy areas, which confirms the redox activity of the solid material, where the inhomogeneity may be a consequence of the random structure of the MVF copolymer. Other oxidants, such as FeCl_3_ or CeIV(SO_4_)_2_, gave similar results. Related features have been observed for spherical assemblies of PVFc block-copolymers [33]. Contact angles for water on MVF films are only marginally decreased by oxidation, and a detailed analysis has not been performed, which would require an investigation of surface roughness, including its oxidation dependence.

## 3. Materials and Methods

### 3.1. General Procedures

All manipulations were performed under an Argon atmosphere unless mentioned otherwise. Prior to use, the glassware was dried in a drying oven at 120 °C. Solvents were distilled over drying agents and subsequently stored under argon atmosphere over 4 Å molecular sieves [34]. Solvents for column chromatography and aqueous workups were used as received (analytical grade supplied by VWR and Alfa-Aesar) without further purification. NMR solvents (purchased from Deutero) were degassed via a few cycles of freeze, pump, and thaw, and finally stored over 3 Å molecular sieves under Argon atmosphere. Reagents and chemicals were purchased from commercial suppliers (Sigma-Aldrich, ABCR, Alfa-Aesar) and used as received. Vinylferrocene, polyferrocenylsilane and polyvinylferrocenes were prepared according to reported procedures [28,30,31].

NMR spectra were measured with Varian 500VNMRS and Varian MR-400 spectrometers at 22 °C. Chemical shifts (δ in ppm) were expressed with respect to TMS (for ^1^H and ^13^C) as standard, set as 0 ppm. The peaks, resulting from the residual non-deuterated NMR solvents, were locked as indicated in the literature [35]. In addition to the standard notation of the signal multiplicity (s = singlet, d = doublet, m = multiplet, dd = doublet of doublet etc.), pst, brs, brd, and brm were used to abbreviate pseudo-triplet, broad singlet, broad doublet, and broad multiplet, respectively in order. For the integration of carbon signals, inverse gated decoupled ^13^C NMR spectra have been recorded using a delay between pulses of 14 s to avoid distortion of the signal intensity owing to incomplete relaxation. Infrared spectra measured for polymers MVF and PMMA were obtained by a Bruker Alpha Platinum ATR spectrometer, where opus 6.5 (by Bruker Optics) was used for analyzing the data. Strong, medium strong, and week peaks for these species have been denoted as s, m, and w, respectively. Elemental analyses were performed without the presence of any external oxidizer (like V_2_O_5_) in an EA 3000 Elemental Analyzer (EuroVector).

All cyclic voltammetry measurements were carried out in an MBraun acrylic glovebox GB2202-C-VAC under Argon atmosphere. Samples were measured as a solution (0.1 M) in dry and deoxygenated CH_2_Cl_2_, where anhydrous [Bu_4_N][PF_6_] was used as a conducting salt at a concentration of 0.1 M. The three-electrode cell consisted of a platinum working electrode, a silver counter electrode, and a silver pseudo reference electrode. The potential was driven on a WaveDriver 20 Bipotentiostat from Pine Research Instrument, and the electrochemical data was recorded via AfterMath (Ver. 1.5.9807, Pine Instrument). All the redox processes were referenced using half-wave potentials of (C_5_Me_5_)_2_Fe as a standard, which was added to the analyzed solution. Its corresponding value was then subtracted from the recorded potentials to convert them to the Fc/Fc^+^ scale following established procedures [36], and finally evaluated with AfterMath and OriginPro.

### 3.2. Synthesis of Poly[(Methyl Methacrylate)-co-(Vinyl Ferrocene)] (MVF)

MVF was synthesized by following a previously-reported synthetic methodology with certain minor improvements [28,29]. A mixture of VFc (4 mmol, 0.848 g), dry and deoxygenated MMA (0.85 mL, 0.800 g, 8 mmol), and azobisisobutyronitrile (AIBN, 0.30 mmol, 0.050 g) was dissolved in dry THF (20 mL). The solution was heated to reflux for 22 h with continuous stirring, filtered, and subsequently precipitated in cold hexanes (0 °C, 200 mL). The precipitates were collected by filtration, redissolved in THF (10 mL), and finally reprecipitated in cold pentane (0 °C, 200 mL). The final precipitate was collected and dried under a high vacuum (10^−3^ mbar) for 24 h before further analyses (yield: 0.486 g (24%) referring to VFc). The filtrates obtained from these precipitation steps were collected and subjected to column chromatography on silica with mixture of hexanes and ethylacetate (1.5:8.5) as eluent to obtain the unreacted VFc. ^1^H NMR (Tol-d8): 1.10–1.50 (brm, -C*H*_3_ of polymerized MMA counterpart), 1.80–2.75 (brm, -CH_2_- of MMA and -CH_2_- and -CH- of polymerized VFc counterpart), 3.15–3.70 (brm, -OC*H*_3_ of polymerized MMA counterpart), 3.75–4.40 (brm, Cp*H* of polymerized VFc counterpart). Inverse gated ^13^C NMR (Tol-d8): 45.25 (d, -*C*H(Me)-CH_2_ of polymerized MMA counterpart), 51.38 (s, -O*C*H_3_ of polymerized MMA counterpart), 69.02 (s, C_5_H_5_ of polymerized VFc counterpart). IR (ATR) *ν*: 1025 (w), 1107 (w), 1143 (s), 1191 (m), 1236 (w), 1435 (w), 1726 (s), 2948 (w), 2988 (w). Anal. calcd for C_26.40_H_35.04_Fe_1.00_O_5.76_ (VFc:MMA = 1:2.88, supporting information file): C, 63.37; H, 7.06. Found: C, 63.47; H, 7.23. Note: For reference, the ^1^H NMR, inverse gated ^13^C NMR (Tol-d8) and ATR-IR of PMMA, synthesized via radical polymerization with AIBN, have been recorded and depicted in a supporting information file.

### 3.3. Imprint Procedure

The thermal and reverse nanoimprint lithography was used to fabricate micro- and nanostructures made of the produced MVF. For this a master template, three different geometries (square, circle, and cross) each in four different sizes (506 nm, 542 nm, 1µm and 3µm) were fabricated in fields of periodic patterns via electron-beam lithography (see Figure 1A). Subsequently, an anti-sticking layer of perfluorodecyltrichlorosilane (FDTS) (Ab111155, ABCR GmbH) was applied to the master template. Therefore, it was placed under a glass dome, and FDTS was evaporated at 250 °C for 1 h. From time to time, the master template was used to fabricate hybrid PDMS stamps. Therefore, the master template was coated with h-PDMS (i), then the h-PDMS layer was covered with s-PDMS, and a glass substrate was used as a mold carrier (ii) (see Figure 1B). The surface of the hybrid PDMS stamp was layered with an anti-adhesive FDTS layer (iii) (see Figure 1C).

The h-PDMS was prepared by mixing two pre-components (A and B) in a ratio of 3:1. Component A (AB112958, ABCR GmbH) and component B (AB171991, ABCR GmbH) were put together and mingled. Afterward, the blend was degassed in vacuo for 20 min. Then, the h-PDMS was applied on the master template and spin-coated for 20 s at 1000 rpm. Subsequently, the coated master template was placed on a hot plate for 20 min at 65 °C.

For the preparation of s-PDMS (Sylgard 184, Dow Corning), base and a curing agent were put together and mixed in a ratio of 10:1. The mixed pre-components were degassed in vacuo for 20 min. Then the s-PDMS was applied on the h-PDMS layer and a glass carrier was placed on top. The carrier was placed 5 mm above the master template with spacers. Afterward, the pre-stamp was heated to 65 °C for 24 h. Finally, the master template and hybrid PDMS stamp were separated mechanically.

As anti-adhesive layer a monolayer of FDTS was applied from the gas phase. First, the surface of the hybrid PDMS stamp was pre-treated with O_2_ plasma (Nano TYP C, Diener Electronic) with 90 W for 1 min. Afterward, the stamp was placed in a desiccator. Then, the desiccator was evacuated, and 0.1 mL FDTS was injected in the desiccator through a septum with a syringe. After 24 h, the desiccator was vented with air, and the stamp was removed and cleaned of excessive FDTS.

For the fabrication of structured MVF layers, a solution containing 100 g/L (poly [(methyl methacrylate)-co-(vinyl ferrocene)]) in toluene was applied on a glass substrate via spin coating (3 s @ 300 rpm; 10 s @ 1000 rpm). Subsequently, the coated substrate was pre-baked for 5 min at 120 °C on a hot plate. Then, the temperature was increased to 170 °C, and the hybrid PDMS stamp was applied onto the co-polymer layer with a pressure of approximately 80 kPa. After 20 min the temperature was decreased to 100 °C and the hybrid PDMS stamp was removed (see Figure 1D).

For the reverse NIL method, a droplet of MVF is applied to our hybrid PDMS stamp at room temperature. Subsequently, it is placed on a glass substrate, which is located on a hotplate at 130 °C, and pressure is applied. After 30 s, the hybrid PDMS stamp with the substrate is removed from the hotplate. After the temperature has dropped to room temperature, the stamp is separated (see Figure 1E).

## 4. Conclusions

In summary, we have synthesized an electrochemically-active vinylferrocene methyl methacrylate co-polymer (MVF) which was used as an imprint polymer for NIL. The characterization of the co-polymer indicates a VFc to MMA ratio of approximately 1:3 based on NMR spectroscopy and combustion analysis. CV measurements of the co-polymer in solution showed reversible redox properties but no electronic interaction between the ferrocene centers. For the NIL process, MVF was dissolved in toluene. Structured surfaces of MVF with squares, circles, and crosses in four different sizes (506 nm, 542 nm,1 µm, and 3 µm) were fabricated via thermal and reverse NIL. Reverse NIL provides the possibility to produce surfaces with patterns of small feature sizes but shows inhomogeneous layer quality over larger areas. In contrast, surfaces fabricated by thermal NIL show better layer homogeneity over large areas, but the transmission of patterns for small feature sizes was incomplete.

In future work, we intend to explore the redox activity and shape-dependent magnetic properties of optimized surface patterns in the oxidized form. As potential applications of nanostructured MVF patterns with large aspect ratios, shape-, and polarity-dependent molecular sorting at interfaces or in channels may be envisioned, where switchable polarity and adhesive properties would be key to reusable sensing or sorting devices.

## Supplementary Materials

The following are available online, Table S1: Determination of vinylferrocene to MMA ratio in MVF, Figures S1–S5: ^1^H and inverse gated ^13^C NMR spectra of co-polymer MVF.
