# Supplementary material for: 3D Micro/Nanopatterning of a Vinylferrocene Copolymer
Source: Molecules. 2020 May 23;25(10):2438. doi: 10.3390/molecules25102438 (PMC7287958; doi:10.3390/molecules25102438)
Supplement: Supplementary file 1 [file molecules-25-02438-s001.pdf]

# Supporting Information

## 3D Micro/Nanopatterning of a Vinylferrocene Copolymer

Dennis Löber, Subhayan Dey, Burhan Kaban, Fabian Roesler, Martin Maurer, Hartmut Hillmer*and Rudolf Pietschnig*

# Table of Contents


**Table S1.** Determination of vinylferrocene to MMA ratio in VFc-MMA-copolymer MVF ........................ 3

**Fig S1.** $^{1}H$ and inverse gated $^{13}C$ NMR NMR of MVF (in Toluene D8, trial 1) .......................................... 4

**Fig S2.** $^{1}H$ and inverse gated $^{13}C$ NMR NMR of MVF (in Toluene D8, trial 2) .......................................... 5

**Fig S3.** $^{1}H$ and inverse gated $^{13}C$ NMR NMR of MVF (in Toluene D8, trial 3) .......................................... 6

**Fig S4.** $^{1}H$ and inverse gated $^{13}C$ NMR NMR of MVF (in Toluene D8, trial 4) .......................................... 7

**Fig S5.** $^{1}H$ (top) and inverse gated $^{13}C$ NMR (bottom) NMR of PMMA (in Toluene D8). .......................... 8

**Fig S6.** ATR-IR spectrum of MVF ............................................................................................................ 9

**Fig S7.** ATR-IR spectrum of PMMA. ........................................................................................... 10

**Table S1.** Determination of vinylferrocene to MMA ratio in VFc-MMA-copolymer MVF

| # Trial of co-polymerization | Trial 1 | Trial 2 | Trial 3 | Trial 4 |
|---|---|---|---|---|
| Vinylferrocene : MMA (by $^1$H NMR) | 1 : 3.2 | 1 : 3.0 | 1 : 2.7 | 1 : 2.8 |
| Avg. Vinylferrocene : MMA (by $^1$H NMR) | 1 : 2.9(2) | | | |
| Vinylferrocene : MMA (by IG $^{13}$C NMR) [a] | 1 : 2.8 | 1 : 2.8 | 1 : 2.8 | 1 : 2.9 |
| Avg. Vinylferrocene : MMA (by IG $^{13}$C NMR) [a] | 1 : 2.8(1) | | | |
| Calculated Formula [VFc : MMA = 1 : 2.8] [b] | $[C_{12}H_{12}Fe]_n[C_5H_8O_2]_{2.8n}$ | | | |
| Calculated % of Carbon | 63.42 | | | |
| Calculated % of Hydrogen | 7.04 | | | |
| % of Carbon (exp. found) | 63.39 | 63.56 | 63.46 | 63.47 |
| % of Hydrogen (exp. found) | 7.10 | 7.17 | 7.32 | 7.33 |
| Avg. % of Carbon (exp. found) | 63.47(1) | | | |
| Avg. % of Hydrogen (exp. found) | 7.23(1) | | | |

[a] Inverse gated $^{13}$C NMR has been termed as IG $^{13}$C NMR in this table.

[b] these values refer just to the ratio of the main chain. Upon including termination with butyronitrile and/or hydrogen changes these values in a range which is still in agreement with the proposed composition and block ratio: $(CH_3)_2$CNC-$[MMA]_3[VFc]$-H, C%: 64.03, H%: 7.45; $(CH_3)_2$CNC-$[MMA]_9[VFc]_3$-H, C%: 63.56, H%: 7.22; $(CH_3)_2$CNC-$[MMA]_{15}[VFc]_5$-H, C%: 63.45, H%: 7.16; $(CH_3)_2$CNC-$[MMA]_{60}[VFc]_{20}$-H, C%: 63.33, H%: 7.10. N.B.: the IR spectra of MVF (Fig. S6) indicate only marginal bands in the region characteristic for alkyl nitriles (2250 to 2230 $cm^{-1}$).

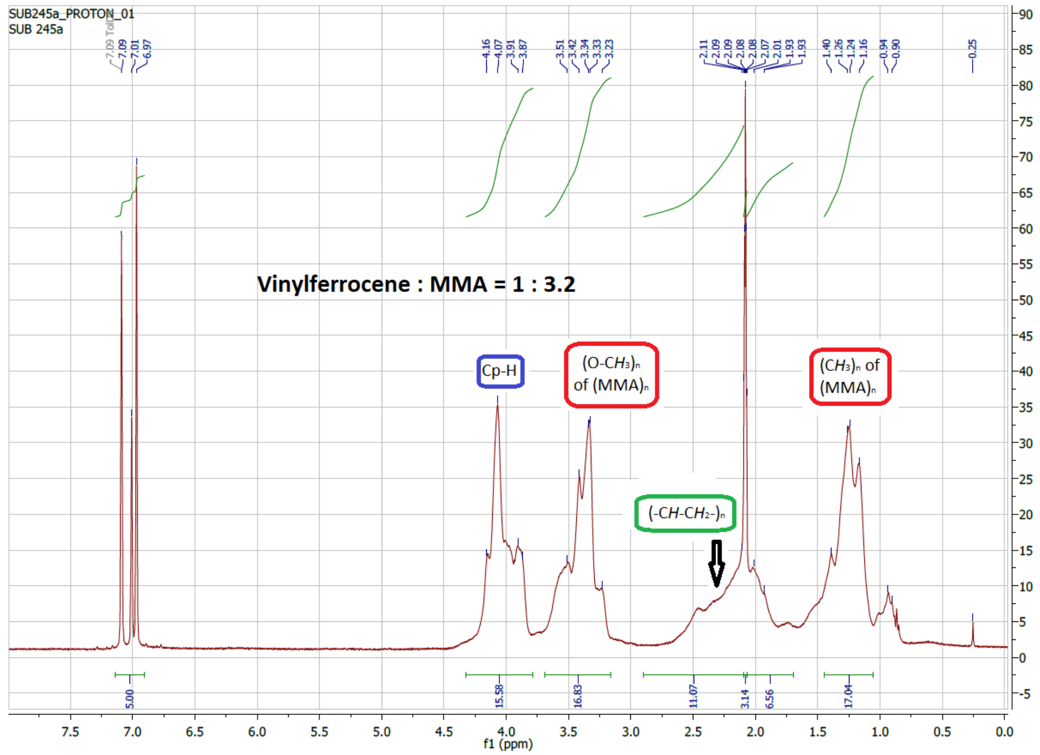


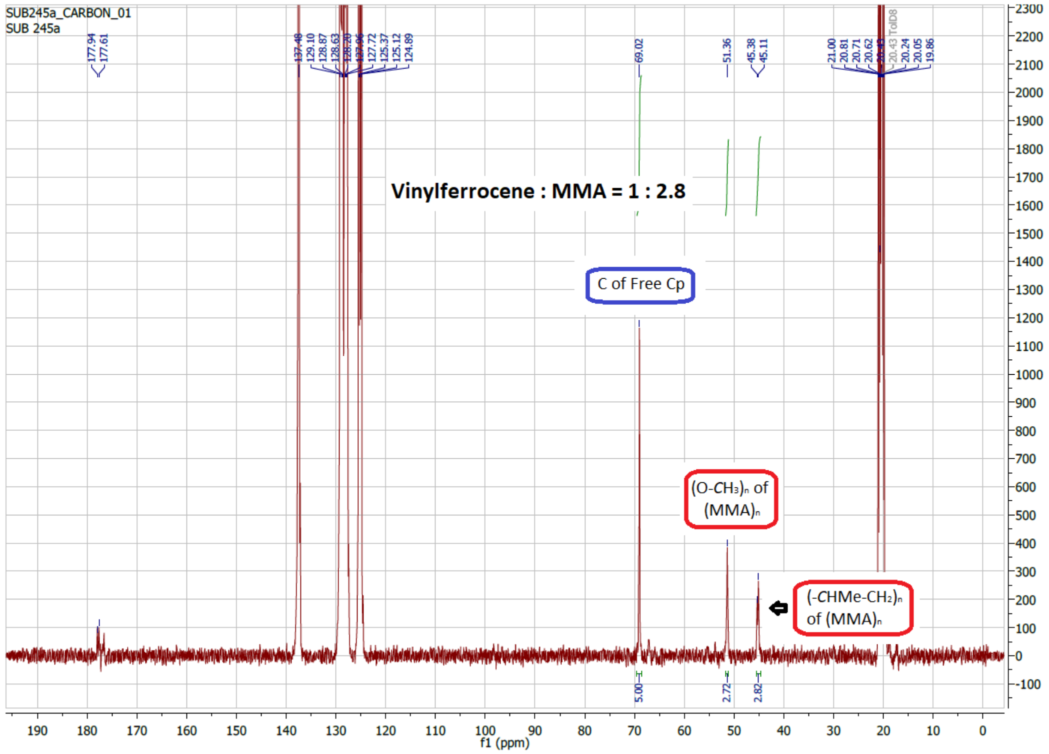


**Fig S1.** $^{1}$H (top) and inverse gated $^{13}$C NMR (bottom) NMR of MVF (in Toluene D8), obtained in trial 1. [Chemical Analysis: Calculated C, 63.37; H, 7.06. Found C, 63.39; H, 7.10.]

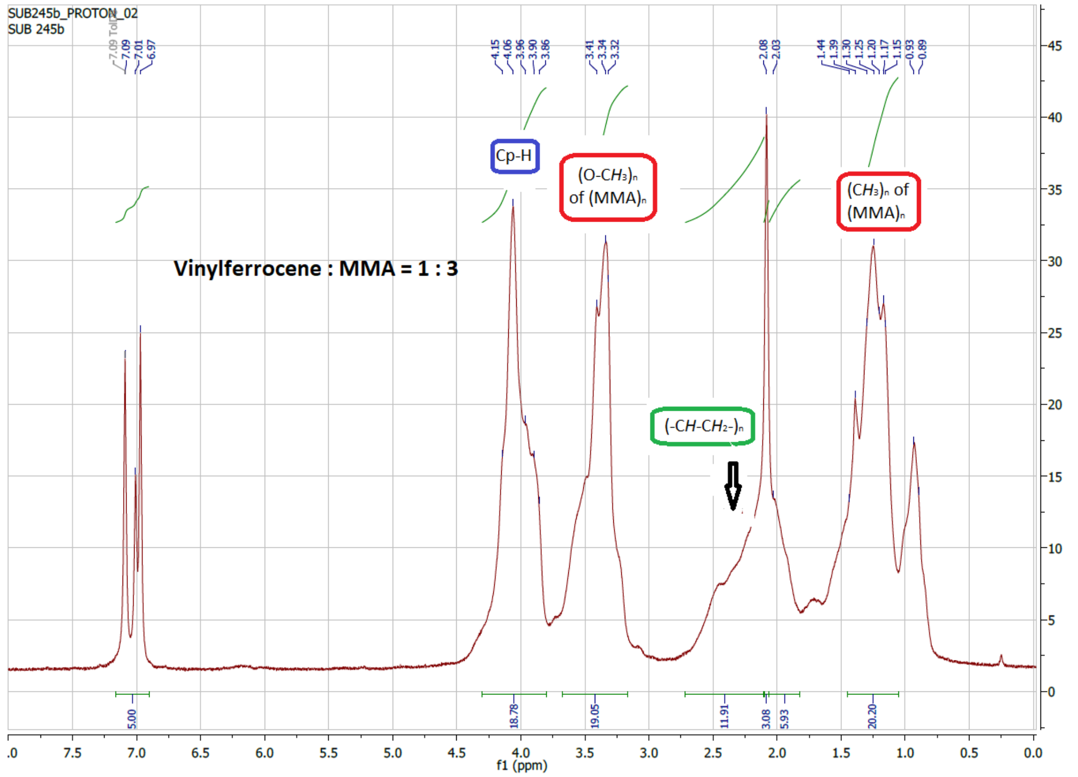


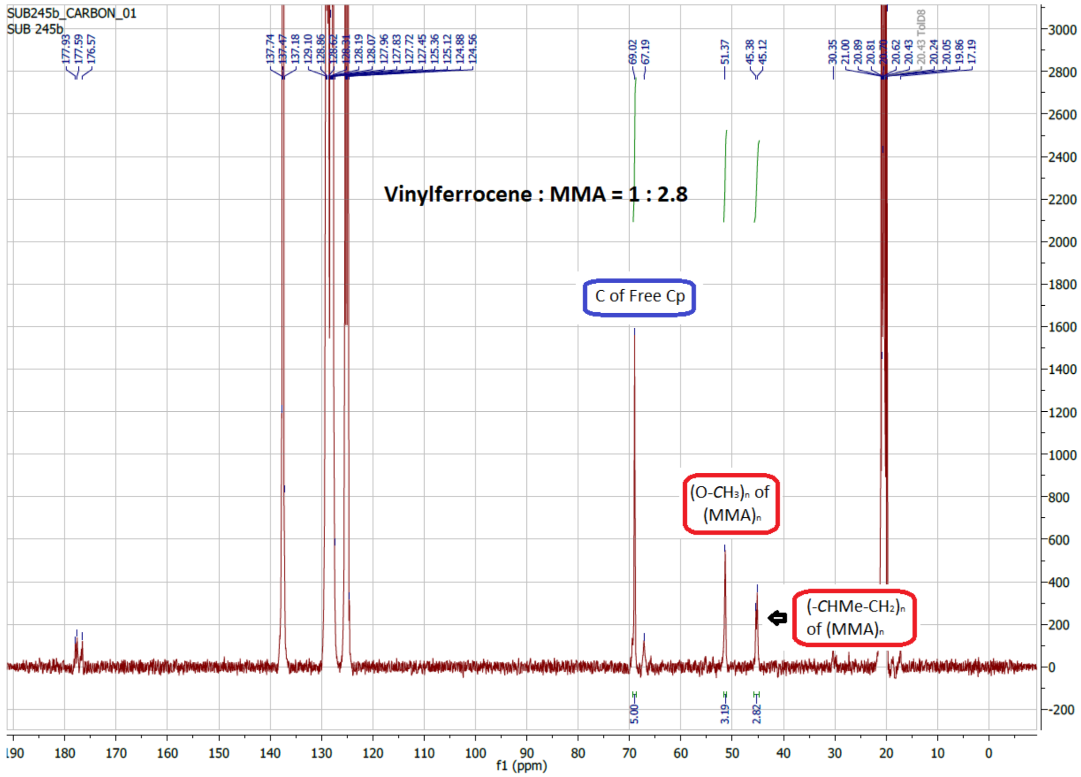


**Fig S2.** $^{1}H$ (top) and inverse gated $^{13}C$ NMR (bottom) NMR of MVF (in Toluene D8), obtained in trial 2. [Chemical Analysis: Calculated C, 63.37; H, 7.06. Found C, 63.56; H, 7.17.]

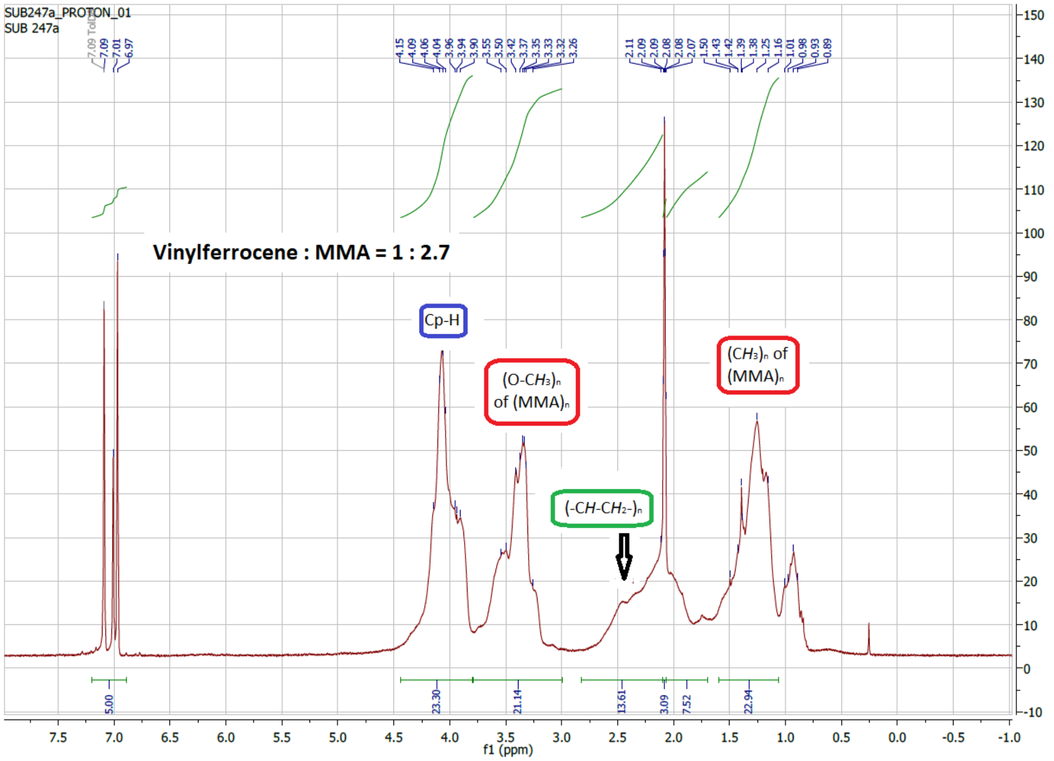


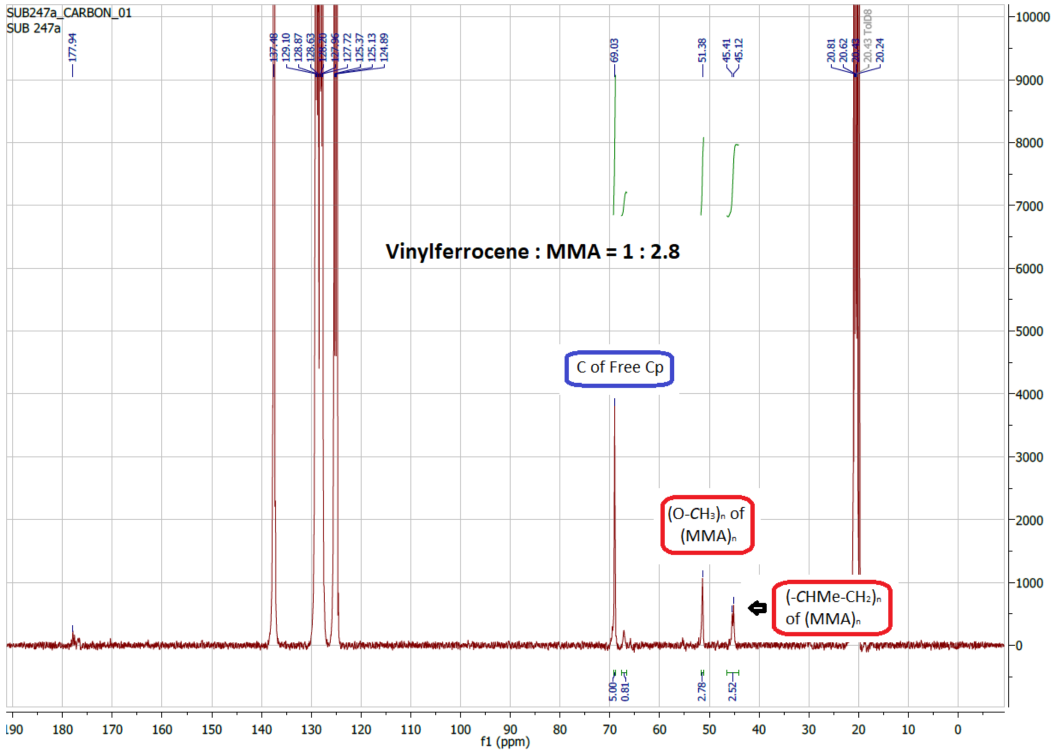


**Fig S3.** $^{1}H$ (top) and inverse gated $^{13}C$ NMR (bottom) NMR of MVF (in Toluene D8), obtained in trial 3. [Chemical Analysis: Calculated C, 63.37; H, 7.06. Found C, 63.46; H, 7.32.]

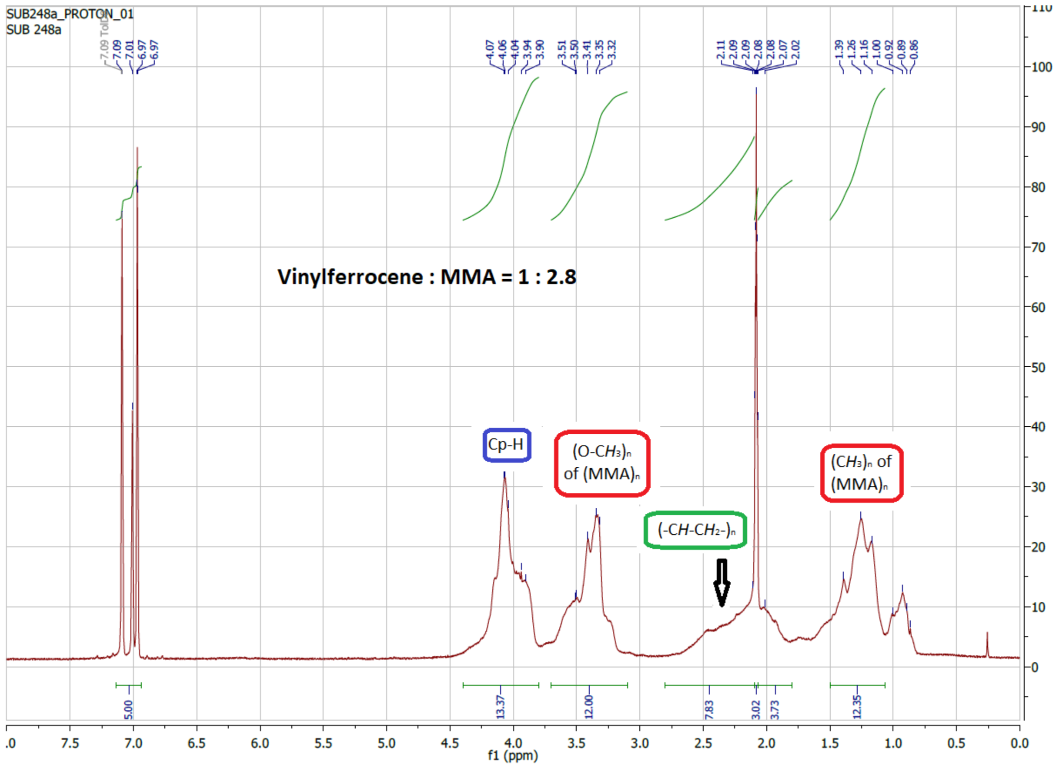


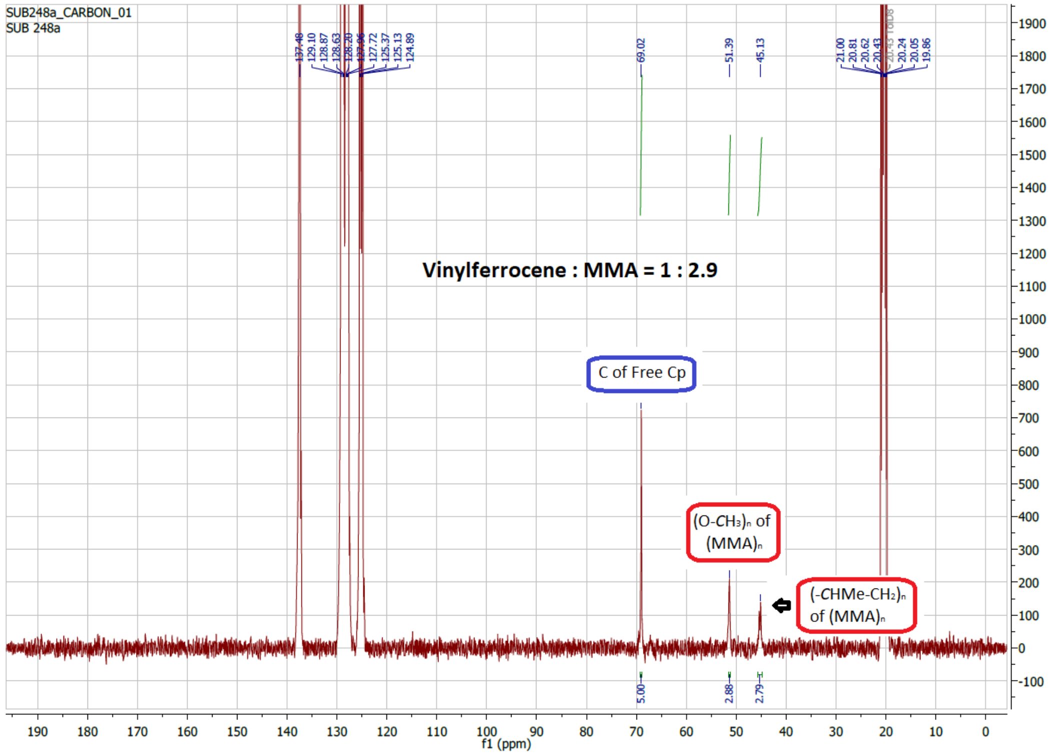


**Fig S4.** $^{1}H$ (top) and inverse gated $^{13}C$ NMR (bottom) NMR of MVF (in Toluene D8), obtained in trial 4. [Chemical Analysis: Calculated C, 63.37; H, 7.06. Found C, 63.47; H, 7.33.]

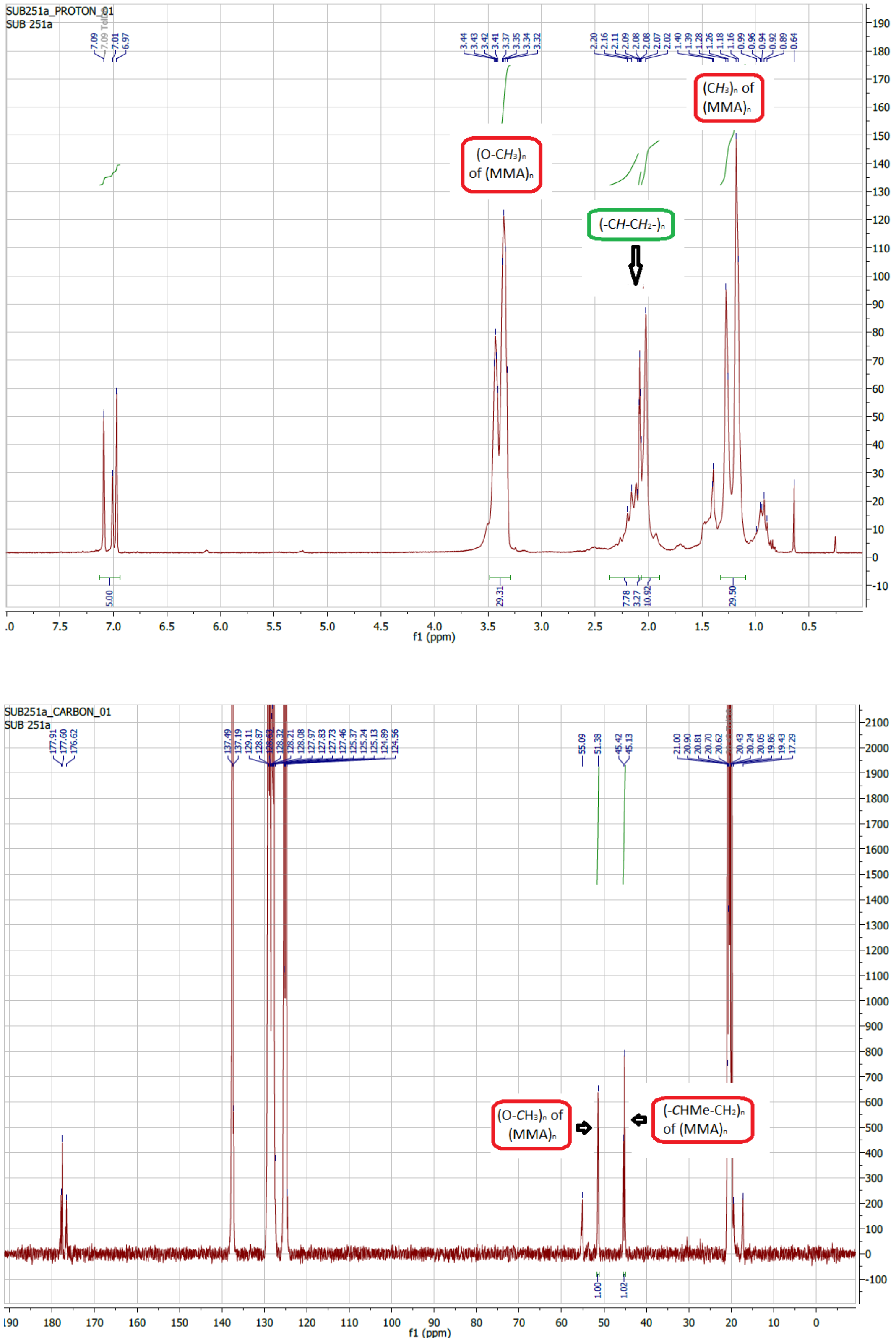


**Fig S5.** $^{1}$H (top) and inverse gated $^{13}$C NMR (bottom) NMR of PMMA (in Toluene D8).

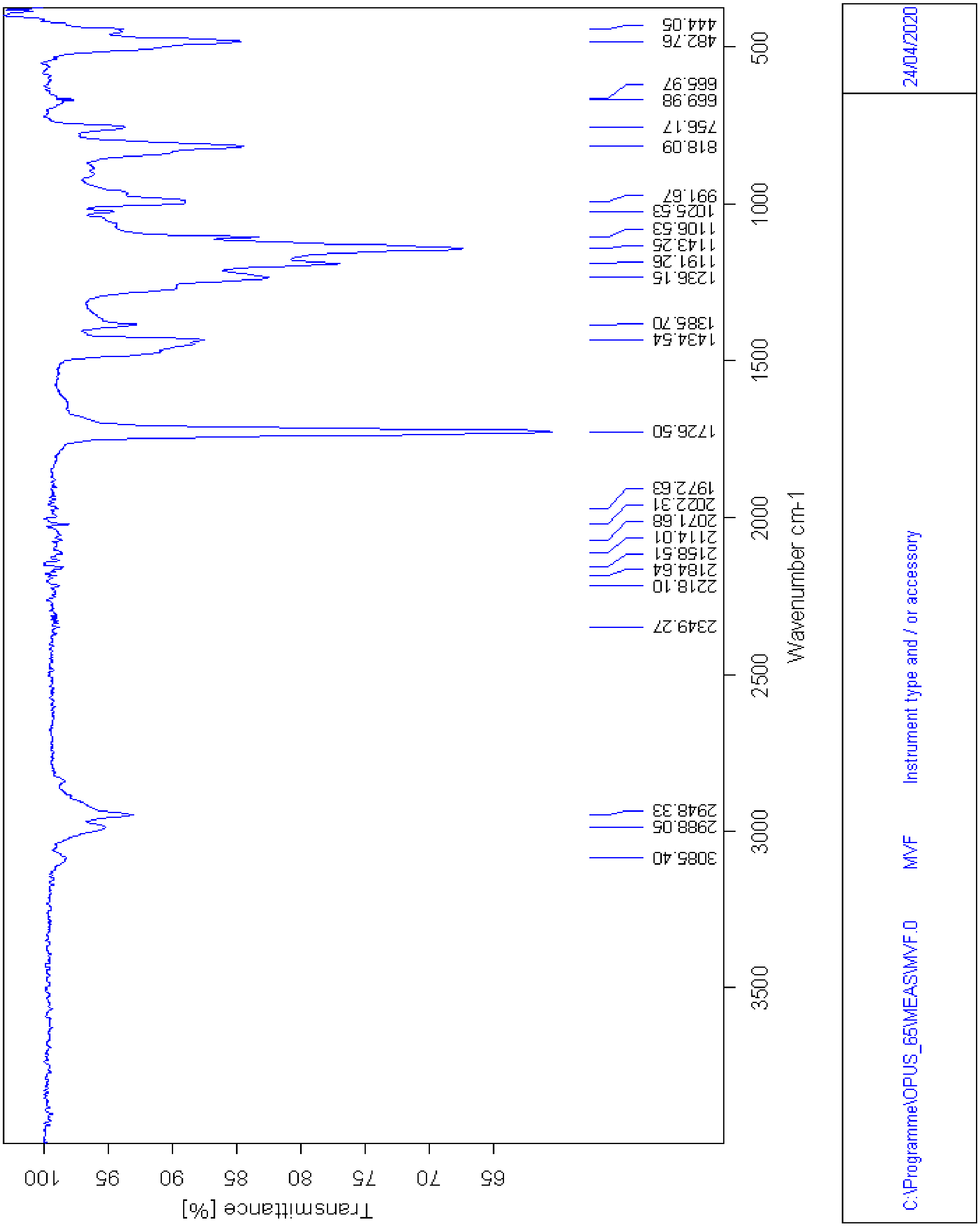


**Fig S6.** ATR-IR spectrum of MVF.

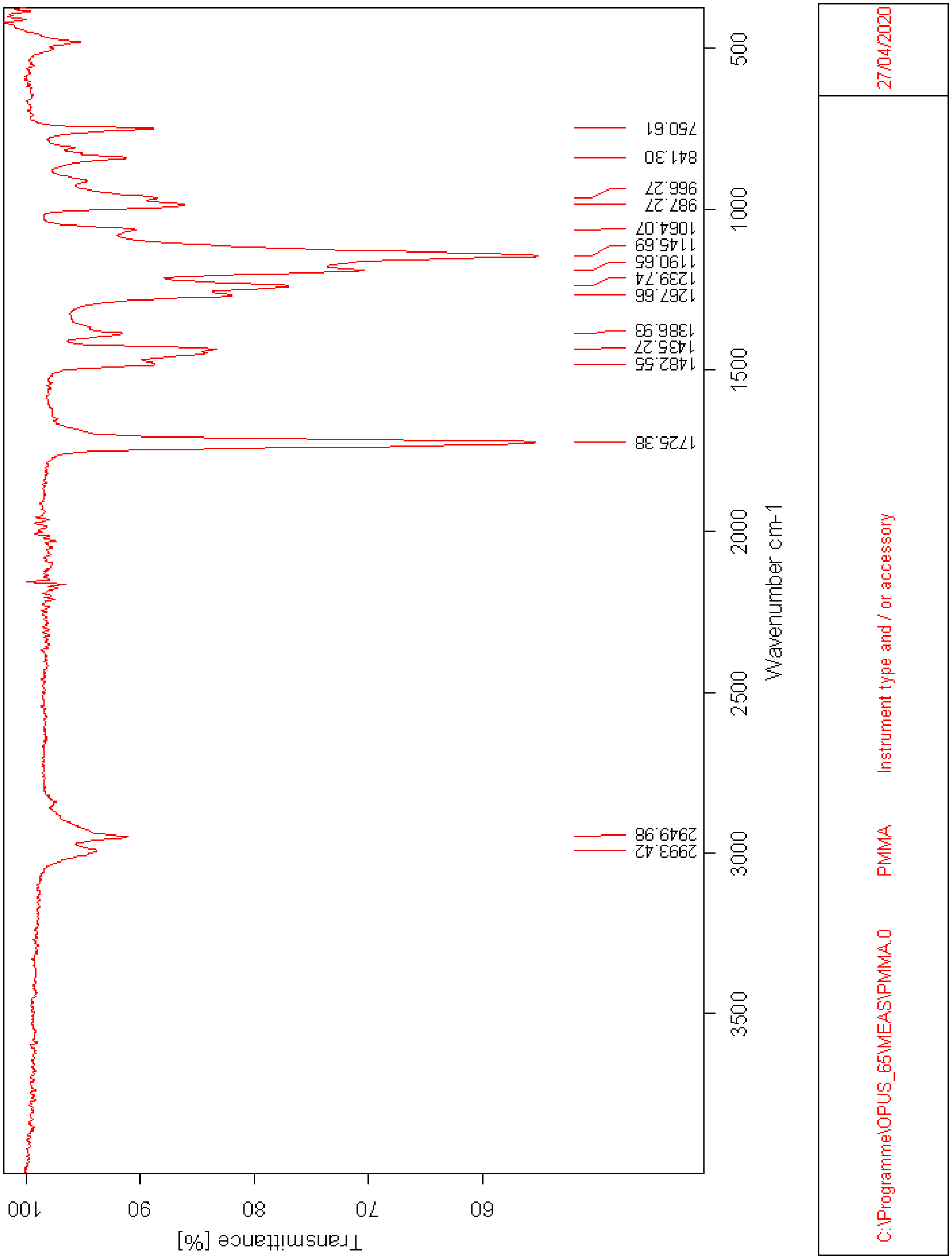


**Fig S7.** ATR-IR spectrum of PMMA.
